# Human amniotic mesenchymal stem cells improve ovarian function in natural aging through secreting hepatocyte growth factor and epidermal growth factor

**DOI:** 10.1186/s13287-018-0781-9

**Published:** 2018-03-09

**Authors:** Chenyue Ding, Qinyan Zou, Fuxin Wang, Huihua Wu, Rulei Chen, Jinghuan Lv, Mingfa Ling, Jian Sun, Wei Wang, Hong Li, Boxian Huang

**Affiliations:** 1 Center of Reproduction and Genetics, Affiliated Suzhou Hospital of Nanjing Medical University, Suzhou Municipal Hospital, Suzhou, 215002 China; 2 Central Laboratory, Affiliated Suzhou Hospital of Nanjing Medical University, Suzhou Municipal Hospital, Suzhou, 215002 China; 3 State Key Laboratory of Reproductive Medicine, Nanjing Medical University, Nanjing, 210029 China

**Keywords:** Natural ovarian aging, Human amniotic mesenchymal stem cells, HGF, EGF

## Abstract

**Background:**

Although many reports show that various kinds of stem cells have the ability to recover function in premature ovarian aging, few studies have looked at stem cell treatment of natural ovarian aging (NOA). We designed this experimental study to investigate whether human amniotic mesenchymal stem cells (hAMSCs) retain the ability to restore ovarian function, and how hAMSCs work in this process.

**Methods:**

To build the NOA mouse model, the mice were fed for 12–14 months normally with young fertile female mice as the normal control group (3–5 months old). Hematoxylin and eosin staining permitted follicle counting and showed the ovarian tissue structure. An enzyme-linked immunosorbent assay was used to detect the serum levels of the sex hormones estradiol (E2), anti-mullerian hormone (AMH), and follicle-stimulating hormone (FSH). The proliferation rate and marker expression level of human ovarian granule cells (hGCs) (ki67, AMH, FSH receptor, FOXL2, and CYP19A1) were measured by flow cytometry (FACS). Cytokines (growth factors) were measured by a protein antibody array methodology. After hepatocyte growth factor (HGF) and epidermal growth factor (EGF) were co-cultured with hGCs, proliferation (ki67) and apoptosis (Annexin V) levels were analyzed by FACS. After HGF and EGF were injected into the ovaries of natural aging mice, the total follicle numbers and hormone levels were tested.

**Results:**

After the hAMSCs were transplanted into the NOA mouse model, the hAMSCs exerted a therapeutic activity on mouse ovarian function by improving the follicle numbers over four stages. In addition, our results showed that hAMSCs significantly promoted the proliferation rate and marker expression level of ovarian granular cells that were from NOA patients. Meanwhile, we found that the secretion level of EGF and HGF from hAMSCs was higher than other growth factors. A growth factor combination (HGF with EGF) improved the proliferation rate and inhibited the apoptosis rate more powerfully after a co-culture with hGCs, and total follicle numbers and hormone levels were elevated to a normal level after the growth factor combination was injected into the ovaries of the NOA mouse model.

**Conclusions:**

These findings provide insight into the notion that hAMSCs play an integral role in resistance to NOA. Furthermore, our present study demonstrates that a growth factor combination derived from hAMSCs plays a central role in inhibiting ovarian aging. Therefore, we suggest that hAMSCs improve ovarian function in natural aging by secreting HGF and EGF.

**Electronic supplementary material:**

The online version of this article (10.1186/s13287-018-0781-9) contains supplementary material, which is available to authorized users.

## Background

The ovary is a major regulator of female reproductive function. Its primary role is to provide a reserve of germ cells established prior to and shortly after birth which gradually decreases the quality and quantity of the oocytes that are contained in the follicles of the ovarian cortex during the period of natural ovarian aging (NOA) [1]. In humans, after the age of 38 years the progressive loss of ovarian follicles accelerates with age. Perimenopause is a midlife transition state experienced by women that results in reproductive senescence [2]. Following the loss of ovarian follicular activity, many perimenopausal symptoms occur, such as vaginal atrophy, osteoporosis, hot flushes, and depression [3].

Delayed childbearing is an important social change that has led to an increasing number of women desiring late menopause. Furthermore, women want to improve their quality of life by avoiding the trouble of perimenopausal symptoms and slowing down ovarian aging. During oocyte development, human granulosa cells (hGCs) play a key role in nurture and support [4]. GCs form the follicular microenvironment which facilitates oocyte development, supplies energy, disposes of waste, and participates in molecular signaling [5]. Research reveals that if the function of GCs becomes impaired with advanced age, oocyte growth and competence are compromised in parallel [6].

Currently, hormonal replacement therapy is used to treat common menopausal problems, but it increases the risk of cancer or recurrence in cancer survivors, forcing physicians to use alternative treatments [7]. Therefore, there is an urgent need to find an effective method to withstand NOA. Recent interest has grown in the therapeutic potential of stem cells, and multipotent stem cells can be developed from different sources such as adipose tissue, bone marrow, and amniotic fluid, all of which show huge therapeutic potential to restore ovarian function and rescue long-term fertility in chemotherapy-treated female mice [8]. Human amniotic mesenchymal stem cells (hAMSCs) present several advantages which make them suitable for clinical therapy: they are an abundant tissue source, show little immunogenicity, are stably proliferative; thus, hAMSCs hold great promise for autologous cell repair and regeneration. AMSCs directly or indirectly (through a paracrine effect, releasing cytokines) participate in the regeneration process [9, 10]. Previous studies show that hepatocyte growth factor (HGF) and epidermal growth factor (EGF) are secreted from multiple mesenchymal stem cells, such as hAMSCs, human amniotic fluid stem cells, and human menstrual blood stem cells [11].

Cytokine EGF is a member of the EGF family and is not only essential for cell proliferation, differentiation, growth, migration, and inhibiting apoptosis, but it also plays an important role in folliculogenesis [12, 13]. A previous study reported that EGF receptor (EGFR) is localized in the follicle cells in both mammals and fish [14, 15]. EGF is reported to increase the proliferation rate of GCs in zebrafish follicles and enhance the cytoplasmic maturation of zebrafish oocytes, which are crucial for oocyte activation, fertilization, and early embryonic development [16]. HGF is a mesenchyme-derived multifunctional protein that plays a critical role in cell survival, proliferation, migration, and differentiation that acts through c-Met receptors [17, 18]. Previous studies suggest that HGF controls numerous key functions which collectively regulate the growth and differentiation of ovarian follicles [19].

Even so, much less is known about that whether hAMSCs retain the ability to resist NOA, and the relevant molecular mechanism of these effects is still not understood. To this end, in the present study, we identified whether hAMSCs maintain the ability to restore ovarian function and how hAMSCs work in this process.

## Methods

### Preparation and culture of hAMSCs

As previously described, hAMSC lines were established in our laboratory [20]. The cells were planted in a 10-cm^2^ culture dish at a density of 1 × 10^7^ in 10 ml of regular growth medium [20] and were incubated at 37 °C and 5% CO_2_. All the experiments were performed with passage 3–4 hAMSCs.

### Establishment of NOA mouse model

Naturally aging female C57BL/6 mice (SPF class, 12–14 months old) and young female C57BL/6 mice with fertility (SPF class, 3–5 months old) were provided by the Nanjing Medical University with Institutional Animal Care and Use Committee approval in accordance with the institutional guidelines. They were bred at a temperature of 28 ± 2 °C with a 12-h light/dark cycle. Vaginal smears of the mice were taken to determine the estrous cycle at 09:00 daily. Only aging mice with disorganized estrous cycles and young mice with normal estrous cycles were chosen. The aging mice were randomly distributed into the control group and cell treatment group (*n* = 15 per group). The young mice were set as the normal control group (*n* = 15 per group).

### Isolation of primary hGCs from NOA patients

Young women (age < 40 years) with tubal occlusion served as the control group (*n* = 31). The NOA patient selection followed a standard that the women were aged > 40 years, had an antral follicle count < 5 or anti-mullerian hormone (AMH) < 1.1 ng/ml and follicle-stimulating hormone (FSH) ≥ 10 mIU/ml (*n* = 26). Women with known normal karyotype, previous chemotherapy or radiotherapy, autoimmune diseases, or ovarian surgery were excluded. Primary hGCs were obtained following informed consent from the normal young women (*n* = 10) and NOA women (*n* = 15) after approval from the Suzhou Hospital Affiliated with the Nanjing Medical University Research Ethics Board. All the participants were treated with recombinant FSH (Puregon; Schering Plough, New Jersey, USA) and gonadotropin-releasing hormone (GnRH) antagonist Ganirelix (Merck, Frosst, Montreal, Canada). Vaginal ultrasound examination was performed to monitor follicular development. Final follicular maturation was induced by administering 10,000 IU of human chorionic gonadotropin (Pregnyl, Merck). hGCs were purified using density centrifugation from follicular aspirates collected from women undergoing oocyte retrieval as previously described [20]. Primary hGCs were cultured in six-well plates in DMEM/F12 media (Thermo, USA) containing 1% penicillin/streptomycin, 10% fetal bovine serum (complete medium), 100 mg/ml streptomycin sulfate (Thermo, USA), and 1× GlutaMAX (Thermo, USA). The culture medium was changed every other day in all the experiments.

### HGF and EGF co-cultured with hGCs and injected into the ovaries of NOA mice

Some of the abovementioned hGCs were divided into the HGF co-cultured group (treatment dose: 90 ng/ml; Sigma), the EGF co-cultured group (treatment dose: 10 ng/ml; R&D system), the growth factor combination (HGF plus EGF) co-cultured group, and the phosphate-buffered saline (PBS) co-cultured control group. After 7 days, hGCs were used for study. The NOA mouse model was divided into the HGF-injected group (treatment dose: 0.9 μg/ml; Sigma), the EGF-injected group (treatment dose: 0.1 μg/ml; R&D system), the growth factor combination-injected group, and a saline-injected control group. After 4 weeks, the mice in each group were killed to evaluate the follicle numbers by an hematoxylin and eosin (H&E) assay.

### Assessment of ovarian function by a comparison of the ovarian follicle count

After cell transplantation, the mice (cell transplanted and non-transplanted) were euthanized from 0 to 4 weeks; the ovaries on two sides were removed and fixed with 10% formalin, paraffin embedded, serially sectioned with a thickness of 5 μm , mounted in order on glass microscope slides, and stained with H&E. Four stage follicles (primordial, primary, secondary, and antral follicles) were detected and classified. The ratio of the number of follicles from the ovary was calculated and compared between each group (per group, *n* = 15). Three representative sections from each ovary were selected. Only follicles containing an oocyte were counted to avoid counting any follicle twice. The experiments were repeated three times, and the results are presented as the fold-change ± SD; *p* < 0.05 determined a significant difference.

### Immunofluorescence staining

The primary antibodies for anti-human-HGF (Abcam, USA), anti-human-transforming growth factor (TGF)-β2 (Abcam, USA), anti-human-EGF (Abcam, USA), anti-human-Osteoprotegerin (Abcam, America), and anti-human-brain-derived neurotrophic factor (BDNF) (Abcam, USA) were selected to characterize ovarian tissues. For the staining procedure, the ovarian sections were fixed with 4% (w/v) paraformaldehyde (PFA; Sigma, USA) at room temperature for 10 min and then washed three times for 5 min with PBS; they were then permeated with 0.1% Triton X-100 (Sigma, USA)/PBS on ice for 10 min and blocked with fresh 4% bovine serum albumin (BSA; Sigma, USA)/PBS at room temperature for 30 min. The treated cells were washed with PBS three times for 5 min and were then incubated with primary antibodies overnight at 4 °C. After rinsing with PBS for 5 min, the cells were stained by Cy2- or FITC-conjugated secondary antibodies (Jackson Immunoresearch, West Grove) in the dark at room temperature for 30 min. The stained cells were mounted with 4′,6-diamidino-2-phenylindole (DAPI; Vector Lab, USA) after washing with PBS for 5 min and were then photographed under a fluorescence microscope (Olympus, Japan).

### Antibody microarray analysis

The cytokines were measured by a protein antibody array methodology (RayBio Human Cytokine Antibody Array, Human Growth Factor Array G1, RayBiotech, Inc., Norgross, GA) that contained antibodies to detect the protein expression level that was differentially expressed in the conditioned media (CM) from hAMSCs. One hundred micrograms of CM were used according to the instructions of the manufacturer.

### Flow cytometry (FACS) analysis

The hGCs were digested separately by trypsin-EDTA for 3 min and were gently blown into single cells which were fixed and permeated by the Cytofix/Cytoper Fixation/Permeabilization Solution Kit (BD, USA) following the instructions of the manufacturer. The hGCs were then stained with PE- or FITC-conjugated antibodies for anti-human-ki67 (Abcam, USA), anti-human-AMH (Thermo, USA), anti-human-FSH receptor (FSHR) (Thermo, USA), anti-human-Forkhead box L2 (FOXL2) (Thermo, USA), anti-human-CYP19A1 (Abgent, USA), and anti-human-Annexin V (Abcam, USA) or their corresponding isotype control for 30 min at 4 °C as described above. These stained cells were analyzed on a fluorescence-activated cell sorter (FACS; Beckman, USA). The experiments were repeated three times, and the results are presented as the fold-change ± SD; *p* < 0.05 determined a significant difference.

### Enzyme-linked immunosorbent assay (ELISA) analysis

Plasma from the NOA mouse model was harvested after hAMSC transplantation or growth factor (EGF, or HGF, or combination) injection to evaluate the expression level of estradiol (E2), AMH, or FSH using an ELISA kit (Mybiosource, USA) according to the manufacturer’s instructions. Briefly, 50 μl of the serum sample was added per well. The test plate was wrapped with a membrane and incubated for 30 min at 37 °C. Thereafter, the wells on the plate were dried and washed with Wash Buffer five times (10 s per wash). Then, 50 μl of the HRP-conjugated reagent was added into each sample well and incubated for 60 min at 37 °C. The samples were washed with Wash Buffer five times (10 s per wash). Subsequently, 50 μl of substrate A Solution followed by 50 μl of substrate B Solution were added and incubated for 15 min at 37 °C. Then, 50 μl of Stop Solution was added into each control and the sample well. Finally, the light absorbance was measured and recorded by a spectrophotometer (Varian Company, Australia).

### Statistical analysis

All the results are shown as means ± SD. Statistically significant differences were determined by a one-way analysis of variance (ANOVA) with SPSS 17.0 software, and *p* < 0.05 was regarded as being statistically significant.

## Results

### hAMSCs restored ovarian function in the NOA mice model

**Fig. 1:**
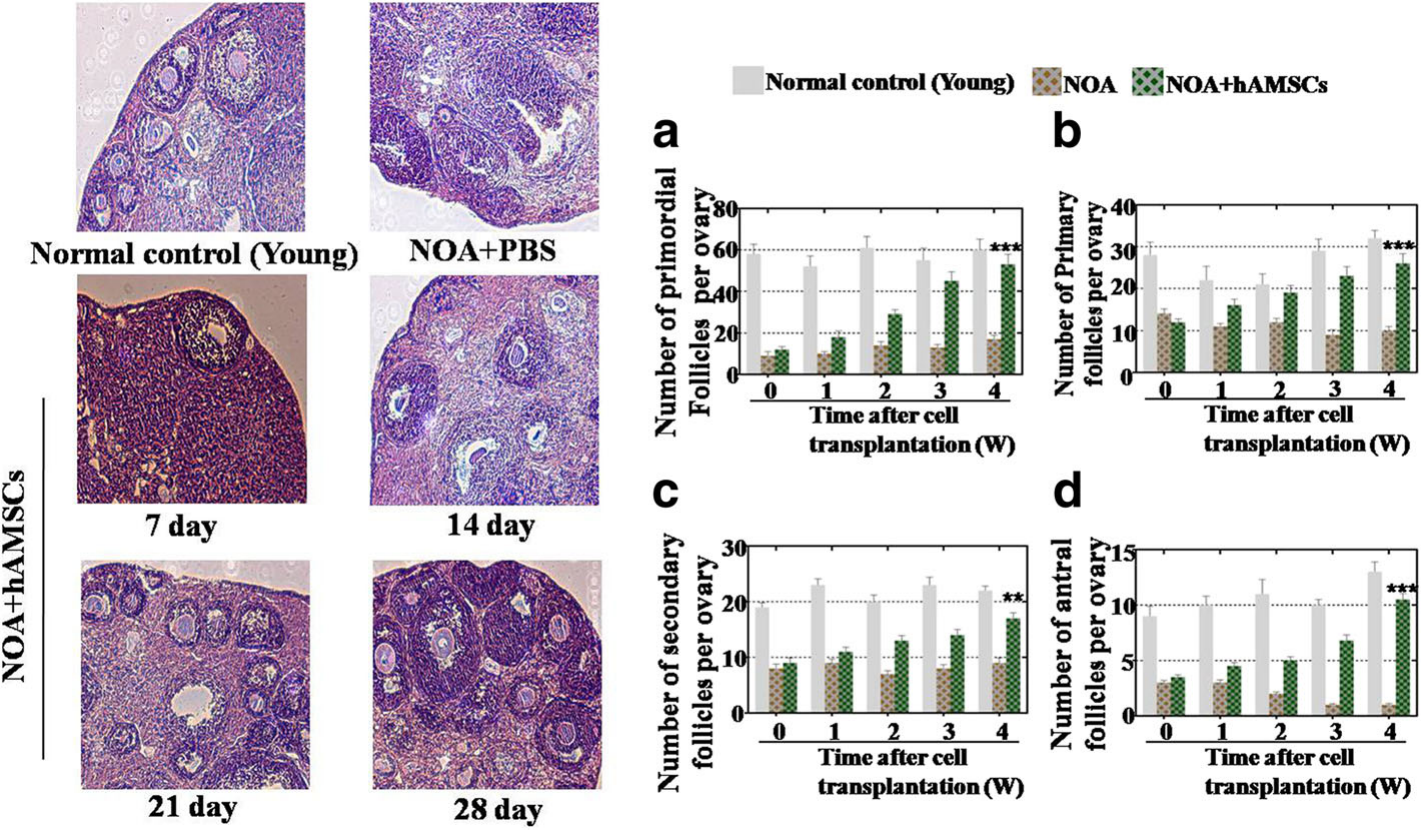
Human amniotic mesenchymal stem cells (hAMSCs) improve the follicle numbers in the ovaries of a natural ovarian aging (NOA) mouse model at four stages. **a** The number of primordial follicles was counted over 4 weeks after hAMSC transplantation. **b** The number of primary follicles was counted over 4 weeks after hAMSC transplantation. **c** The number of secondary follicles was counted over 4 weeks after hAMSC transplantation. **d** The number of antral follicles was counted over 4 weeks after hAMSC transplantation. All the experiments were repeated three times; the error bars indicate SD. **p < 0.01, ***p < 0.001, versus the NOA group. PBS phosphate-buffered saline

First, the hAMSC lines were established successfully as described in a previous paper [20]. After the hAMSCs were injected into the ovaries of the NOA mice, the H&E stained ovarian tissues showed that the hAMSCs restored the follicle numbers to 21% for primordial follicles, 42% for primary follicles, 58% for secondary follicles, and 46% for antral follicles at week 1 compared with the levels in the control group (Fig. 1). In contrast, after the hAMSCs were injected for 4 weeks, our results showed that the hAMSCs recovered the follicle numbers to 88% for primordial follicles, 89% for primary follicles, 86% for secondary follicles, and 81% for antral follicles compared with the levels in the control group which was a significant improvement (*p* < 0.01) compared with the NOA group (Fig. 1).

**Fig. 2:**
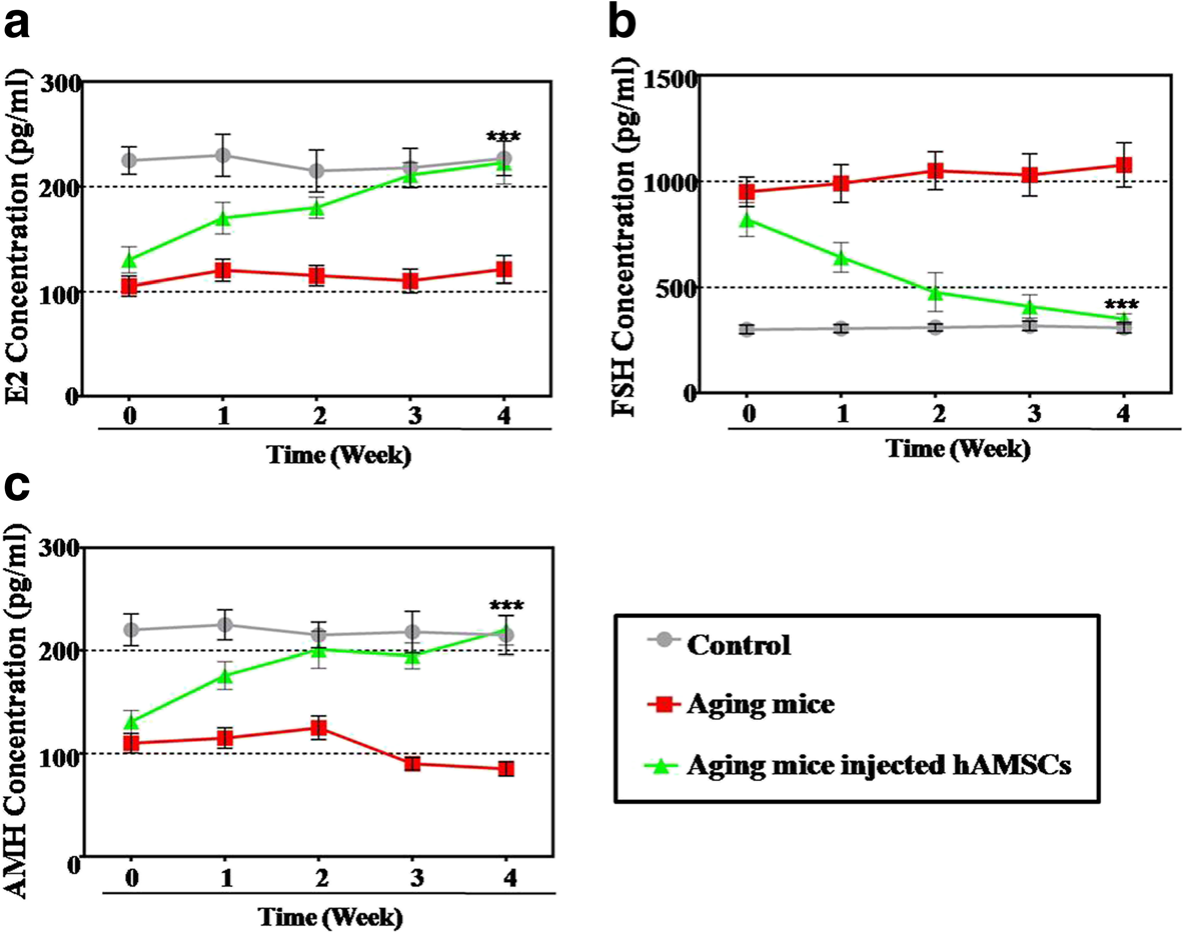
Human amniotic mesenchymal stem cells (hAMSCs) improve the hormone levels in the ovaries of an NOA mouse model. **a** The level of estradiol (E2) was measured by ELISA over 4 weeks after hAMSC transplantation. **b** The hormonal level of follicle-stimulating hormone (FSH) was measured by ELISA over 4 weeks after hAMSC transplantation. **c** The hormonal level of anti-mullerian hormone (AMH) was measured by ELISA over 4 weeks after hAMSC transplantation. All the experiments were repeated three times; the error bars indicate SD. ***p < 0.001, versus the aging mice group

The hormone levels of the plasma in each group was tested after hAMSC transplantation. In the treatment group, an ELISA assay demonstrated that the level of E2 (72%) and AMH (77%) was slightly rescued compared to the aging group (50% E2 and 52% AMH) and to that of the control group at week 1 (Fig. 2a, c). However, at week 4, the hAMSCs rescued the level of E2 (95%) and AMH (92%) to the normal levels of the control group which was a significant improvement (*p* < 0.001) compared with the NOA group (Fig. 2a, c). In addition, the level of FSH decreased to 209% of that in the treatment group compared to 325% in the aging mice group at week 1. In contrast, after hAMSC treatment for 4 weeks, the level of FSH was rescued to the normal control group level (109%) which was a significant improvement (*p* < 0.001) compared with the NOA group (Fig. 2b).

Overall, the hAMSCs exhibited a powerful ability to restore ovarian function in an NOA mouse model. 

### hAMSCs increased the proliferation rate and marker expression of the hGCs

**Fig. 3:**
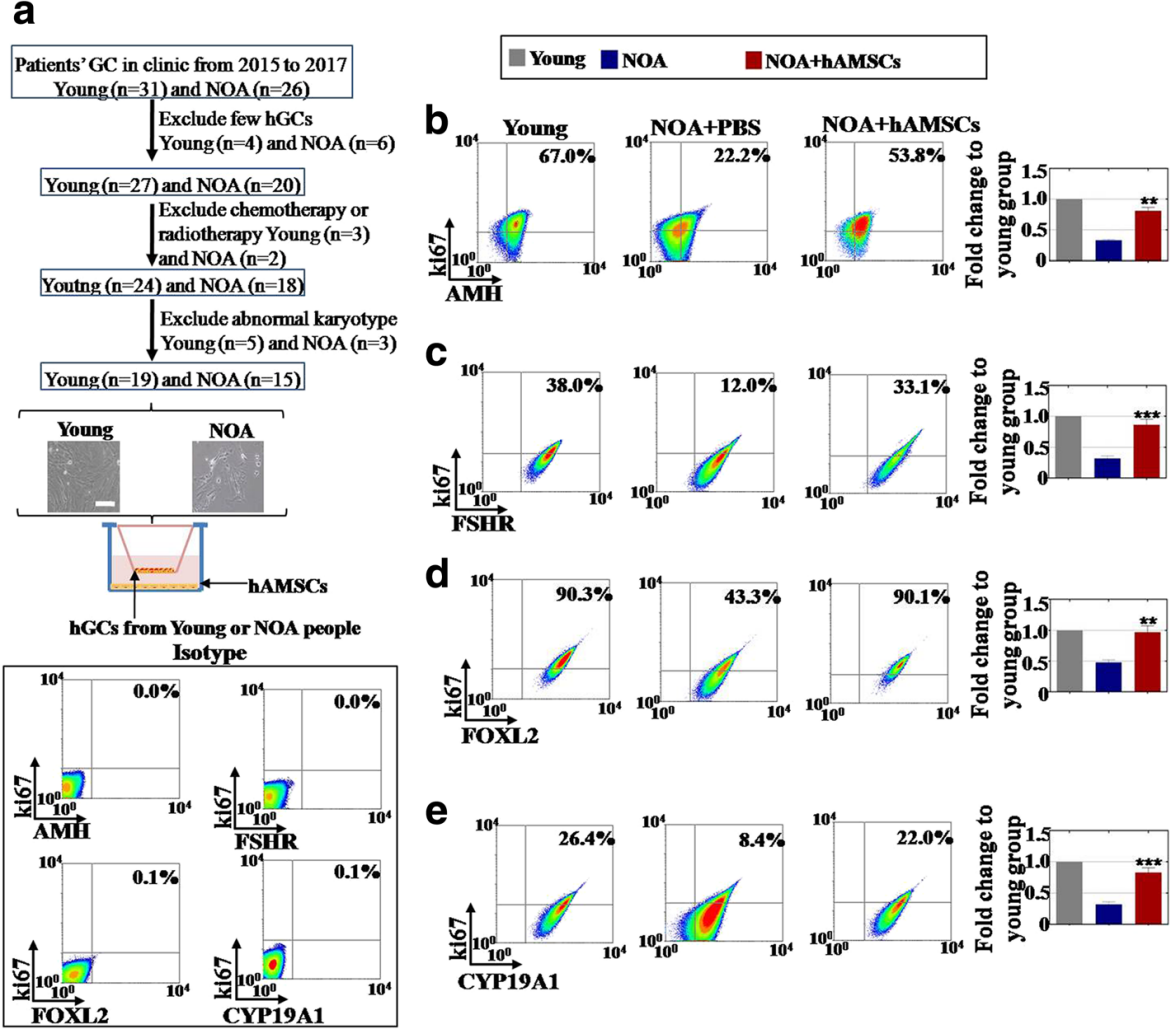
Human amniotic mesenchymal stem cells (hAMSCs) improve the proliferation rate of human granulosa cells (hGCs) and upregulate the expression of hGC markers. **a** A schematic overview of the hGC filter procedures. **b** The number of ki67^+^AMH^+^ hGCs was evaluated after a co-culture with hAMSCs. **c** The expression level of the ki67^+^FSHR^+^ hGCs was tested after a co-culture with hAMSCs. **d** The expression level of the ki67^+^FOXL2^+^ hGCs was tested after a co-culture with hAMSCs. **e** The number of ki67^+^CYP19A1^+^ hGCs was evaluated after a co-culture with hAMSCs. The experiments were carried out after 7 days of co-culture, *n* = 3; error bars indicate SD. **p < 0.01, ***p < 0.001, versus the NOA group. AMH anti-mullerian hormone, FOXL2 forkhead box L2, FSHR follicle-stimulating hormone receptor, NOA natural ovarian aging, PBS phosphate-buffered saline

To investigate the therapeutic effects of hAMSCs in NOA patients in the clinic, we collected hGCs from a young group (*n* = 19) and an NOA patient group (*n* = 15) in our reproductive center to examine the effects of cell proliferation after co-culturing with hAMSCs (Fig. 3a). hGC markers (AMH, FSHR, FOXL2, and CYP19A1) and a ki67 antibody (cell proliferation marker) were used to estimate the effects from hAMSCs by a FACS analysis method. Our results showed that hAMSCs increased the ki67^+^AMH^+^ cell number in the NOA group to a higher level (81%, *p* < 0.001) than the PBS administration group (33%) compared with the young group (Fig. 3b). The FACS assay results demonstrated that hAMSCs increased the ki67^+^FSHR^+^ cell number to 86% (*p* < 0.001) in the NOA group, higher than the 32% in the PBS administration group compared with that of the young group (Fig. 3c). Our results further revealed that hAMSCs increased the ki67^+^FOLX2^+^ cell number in the NOA group to a greater degree (97%, *p* < 0.001) than the 48% seen in the PBS administration group compared with that of the young group (Fig. 3d). In addition, the FACS assay results showed that the hAMSCs raised the ki67^+^CYP19A1^+^ cell number to 83% (*p* < 0.001) in the NOA, higher than the 32% in the PBS co-cultured group compared with that of the young group (Fig. 3e).

Overall, the hAMSCs showed powerful recovery effects for natural senescent hGCs.

### hAMSC-derived HGF and EGF levels were higher than other growth factors

**Fig. 4:**
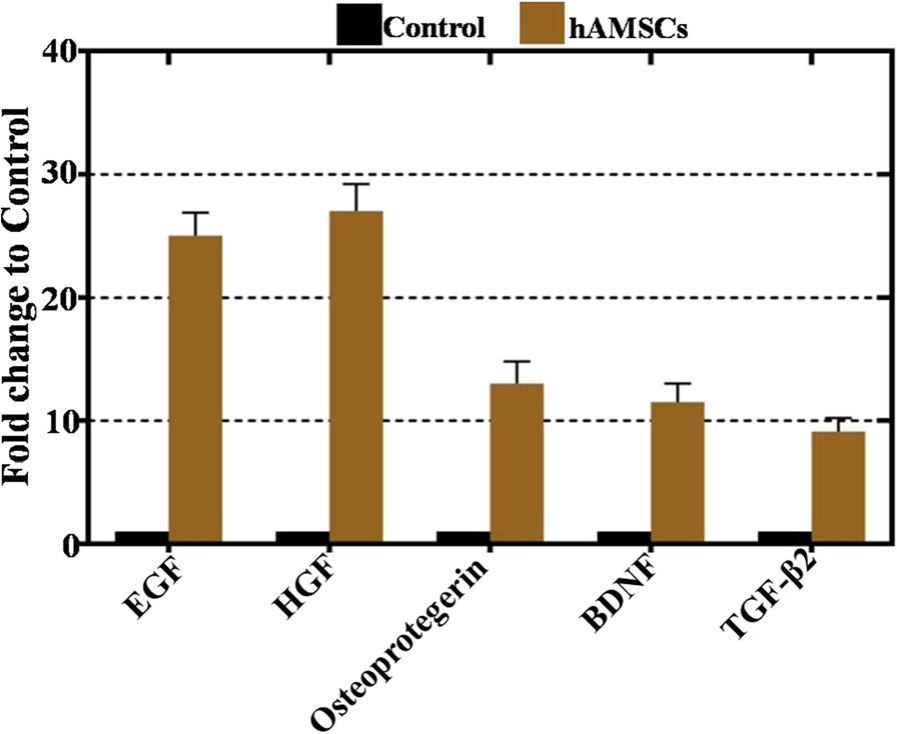
Epidermal growth factor (EGF) and hepatocyte growth factor (HGF) derived from the human amniotic mesenchymal stem cells (hAMSCs) were higher than other growth factors. HGF and EGF derived from the hAMSCs exhibited a higher level of secretion than other growth factors. All the experiments were repeated three times; error bars indicate SD. BDNF brain-derived neurotrophic factor, TGF transforming growth factor

**Fig. 5:**
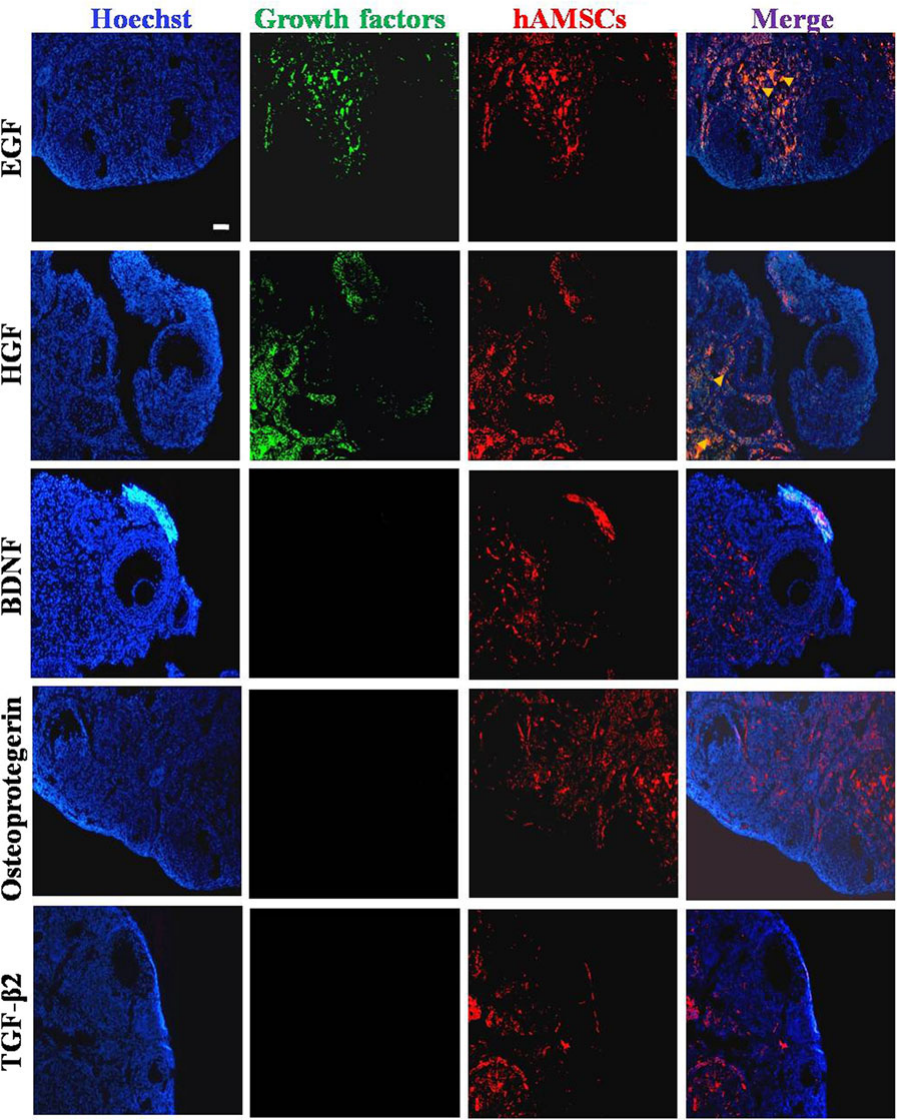
Immunofluorescence analysis of the cytokines hepatocyte growth factor (HGF), epidermal growth factor (EGF), brain-derived neurotrophic factor (BDNF), Osteoprotegerin, and transforming growth factor (TGF)-β2 in the ovaries after human amniotic mesenchymal stem cell (hAMSCs) transplantation. HGF and EGF were highly expressed in the ovary tissue of the NOA mice after hAMSC transplantation

To investigate the mechanism of the restoring effect of transplanting hAMSCs into NOA mice, the supernatant was collected from three hAMSC lines derived from three different donors (two male (hAMSCs-04, -05), one female (hAMSCs-01)) and the control group (293 T cell line), and then a cytokine antibody array (growth factors = 52) was utilized to evaluate the diversity. Our analytical data elucidated that the large number of growth factors in the hAMSCs exceeded the control group (Additional file 1: Figure S1A). There were 23 growth factors from the hAMSCs that were secreted at a significantly higher level than in the control group (*p* < 0.05) (Additional file 1: Figure S1B). In accordance with standard criteria of fold-change greater than or equal to 8 and statistical significance (*p* < 0.01), five growth factors were further selected: BDNF, EGF, Osteoprotegerin, HGF, and TGF-β2 (Additional file 1: Figure S1B). The secretion level of EGF and HGF was obviously higher than the other growth factors (Fig. 4). After the hAMSCs were transplanted into the NOA mice for 4 weeks, an immunofluorescence assay revealed that EGF and HGF were expressed in ovaries of the NOA mice, but not BDNF, Osteoprotegerin, or TGF-β2 (Fig. 5).

Taken together, these results suggest that EGF and HGF might play a vital role in improving ovary function of natural aging mice.

### HGF and EGF combined improved the proliferation rate and inhibited the apoptosis rate in hGCs

**Fig. 6:**
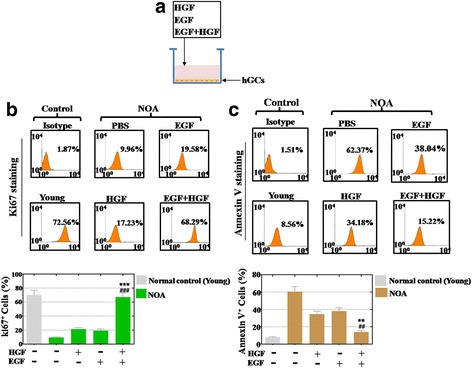
A combination of epidermal growth factor (EGF) and hepatocyte growth factor (HGF) improves proliferation and inhibits the apoptosis level to a greater degree than using them alone after a co-culture with human granulosa cells (hGCs). **a** A schematic diagram of the EGF and HGF co-culture with hGCs. **b** A combination of EGF and HGF improved proliferation in the hGCs more significantly than using them singly. **c** A combination of EGF and HGF inhibited apoptosis in the hGCs more effectively than using them singly. All the experiments were repeated three times; error bars indicate SD. **p < 0.01, ***p < 0.001, versus the HGF group; ^##^*p* < 0.01, ^###^*p* < 0.001, versus the EGF group. NOA natural ovarian aging, PBS phosphate-buffered saline

To examine the effects of HGF and EGF on cell proliferation and apoptosis, hGCs were co-cultured with HGF and EGF for 7 days (Fig. 6a). A FACS assay was employed to quantitatively assess cell viability. Our results showed that the proliferation rate of the hGCs was raised to 21% in the HGF group and 19% in the EGF group, which was higher than the untreated group (9%), compared with that of the young normal control mice group at 70% (Fig. 6b). The FACS assay also showed that the growth factor combination group (HGF plus EGF) increased the proliferation rate of the hGCs to a higher degree (reaching 67%), significantly higher than the HGF-alone and EGF-alone groups (*p* < 0.001, Fig. 6b). Similarly, the results from the apoptosis assay indicated that a single growth factor decreased the rate of apoptosis to 34% in the HGF group and 38% in the EGF group, which was lower than the untreated group (61%), compared with of the young mice group at 8% (Fig. 6c). The combined group of growth factors inhibited the rate of apoptosis more strongly at 14% (*p* < 0.01), significantly different from the HGF and EGF groups (Fig. 6c).

In summary, a combination of two growth factors increased the proliferation rate and inhibited the apoptosis rate of hGCs more effectively than using them singly.

### The HGF and EGF combination group increased the follicle number and improved the hormone level in NOA mice

**Fig. 7:**
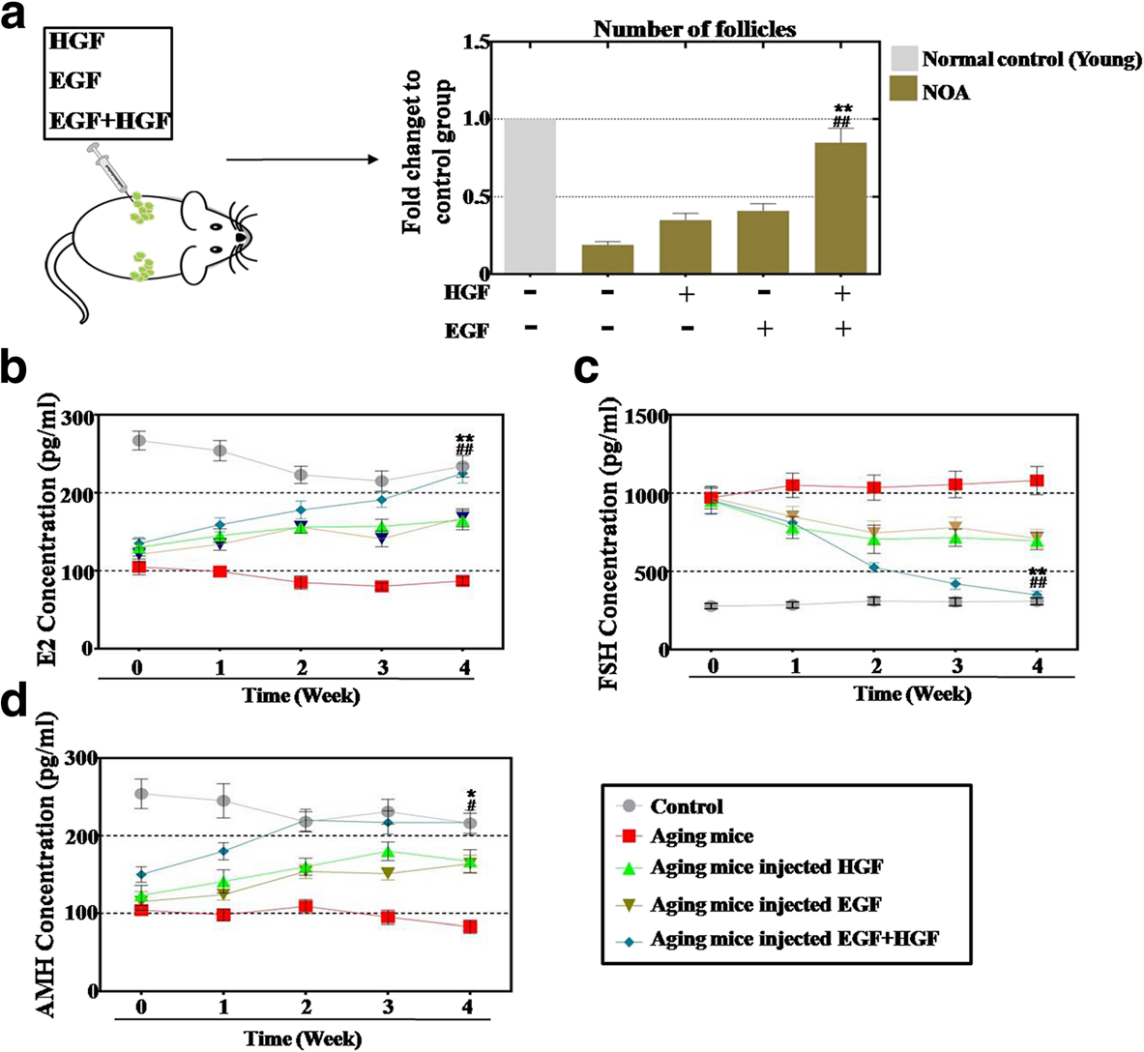
A combination of epidermal growth factor (EGF) and hepatocyte growth factor (HGF) increases the follicle numbers and elevates the hormone levels to a greater extent than using them alone after an injection into a natural ovarian aging (NOA) mouse model. **a** A combination of EGF and HGF increased the follicle numbers to a higher level than using them singly. **b** A combination of EGF and HGF increased the level of estradiol (E2) to a higher level than using them singly. **c** A combination of EGF and HGF increased the level of follicle-stimulating hormone (FSH) to a higher level than using them singly. **d** A combination of EGF and HGF increased the level of anti-mullerian hormone (AMH) to a higher level than using them singly. All the experiments were repeated three times; error bars indicate SD. *p < 0.05, **p < 0.01, versus the HGF group; ^#^*p* < 0.05, ^##^*p* < 0.01, versus the EGF group

The follicle numbers were counted after the HGF and EGF injection into the ovaries of a natural aging mouse model. Our result showed that using the growth factors singly increased the number of follicles to 35% in the HGF group and 41% in the EGF group, which was a little more than the natural aging group (19%) compared with that in the young mice group (Fig. 7a). After the growth factor combination was injected into the ovaries of the NOA mice, we found that the number of follicles increased to 85% of that in the young control mice, which was significantly different to the values in the HGF and EGF single groups (*p* < 0.01) (Fig. 7a). Furthermore, the hormone levels in the plasma in each group were also determined after HGF and EGF injections. The ELISA assay results revealed that the level of E2 and AMH increased moderately in the HGF group (reaching 52% and 55% of the value in the control group), the EGF group (reaching 49% and 48% of the value in the control group), and the growth factor combination group (reaching 59% and 68% of the value in the control group), which was moderately higher than the untreated group (reaching 40% and 42% of the value in the control group) after injections for 1 week (Fig. 7b, d). However, after HGF and EGF injection for 4 weeks, the level of E2 and AMH increased to 68% and 72% of the value in the EGF group, and 69% and 73% of the value in the HGF group. In contrast, the growth factor combination rescued the levels of E2 and AMH to normal (98% and 99% of the value in the control group), with no improvement seen in the young mice group. The combination injection was significantly higher than the single growth factor injections (*p* < 0.01 for E2; *p* < 0.05 for AMH) (Fig. 7b, d). This finding was also seen for FSH. Our results showed that the level of FSH increased to 273% in the HGF group, 298% in the EGF group and 284% in the growth factor combination group, results which were a little lower than that of the aging group (369%), compared with that of the young mice group after an injection for 1 week (Fig. 7c). However, after HGF and EGF injection for 4 weeks, a single growth factor inhibited the levels of FSH to just 237% in the HGF group and 248% in the EGF group compared to the young mice group, whereas the growth factor combination rescued the level of FSH to normal levels (98% of the control group) compared to the young mice group at week 4, significantly lower than either single growth factor injection group (*p* < 0.01) (Fig. 7c).

Taken together, these results suggest that the growth factor combination group played a vital role in improving ovary function in a natural aging mouse model.

## Discussion

Various types of stem cells have been used to restore premature ovarian failure, including human amniotic epithelial cells, bone marrow mesenchymal stem cells, human amniotic fluid stem cells, and human menstrual blood stem cells [11]. However, it is still not understood whether hAMSCs retard NOA and how hAMSCs work. In this study, after hAMSC transplantation, the follicle numbers at four stages were nearly recovered to normal levels in the NOA mice. We analyzed the serum index of E2, AMH, and FSH, and all these were restored to normal levels (Figs. 1 and 2). In addition, to bridge the bench-to-bedside gap, the preclinical efficacy of hAMSCs in delaying NOA were evaluated, and hGCs that derived from NOA patients were co-cultured with hAMSCs. Our findings indicate that hAMSCs increased the proliferation rate and marker expression of hGCs to natural levels (Fig. 3). Our research, therefore, firstly confirmed that hAMSCs have ability to withstand NOA.

Although data from several studies suggest that hAMSCs plays a pivotal role in wound healing, anti-neural aging, and angiogenesis [9, 10, 21], few studies reveal the mechanisms by which hAMSCs achieve these results. Specifically, little is known about the mechanism of how hAMSCs resist NOA. According to a previous reports, AMSCs withstand premature aging and reveal an antioxidant ability via secretory cytokines [22–24]. In our results, antibody microarray results revealed that growth factor HGF and EGF secretions from hAMSCs were expressed at a high level (Fig. 4). Meanwhile, after hAMSC transplantation, HGF and EGF were highly expressed in the ovaries of natural aging mice (Fig. 5). Several lines of evidence support that higher levels of HGF and EGF are secreted by mesenchymal stem cells [22, 25].

Even so, there is no reliable evidence whether HGF and EGF function to withstand NOA. To address this issue further, a growth factor combination group was used. After a growth factor combination was added to the hGCs, our FACS results revealed that HGF plus EGF improved the proliferation rate and reduced the apoptosis rate of the hGCs more robustly than using either factor alone (Fig. 6). Furthermore, an ovarian function study also showed similar results. When HGF and EGF were injected into the ovaries of natural aging mice together, the total follicle number and the hormone levels were elevated more notably than by using a single growth factor (Fig. 7). Moreover, earlier research also reveals that EGF-like growth factors play a critical role in peri-ovulatory events in humans [14]. HGF is a known crucial regulator of anti-apoptosis in preantral follicles [26]. These current findings are consistent with results from a previous study showing that HGF and EGF promote cell proliferation and delay oocyte aging during culture in vitro [27, 28].

## Conclusions

We investigated the interaction between hAMSCs and NOA for the first time. The results of the present study provide insight into the notion that hAMSCs delay NOA by secreting HGF and EGF. Furthermore, our present study revealed that a growth factor combination derived from hAMSCs plays a central role in inhibiting ovarian aging. Therefore, we suggest that hAMSCs improve the ovarian function of natural aging by secreting HGF and EGF. This discovery has important implications for understanding the molecular mechanisms by which growth factors promote the ovarian function in natural aging. Moreover, this discovery suggests that HGF and EGF may serve as a novel, safe, and efficacious therapeutic schedule to resist NOA and improve female reproductive health.

## Additional file

Additional file 1: **Figure S1.** An antibody microarray to test the level of the growth factors derived from the hAMSCs. (A) Antibody microarray analysis of the growth factor secretion from the hAMSCs and the control group. (B) Five growth factors were selected in accordance with the standard criteria that the fold-change was greater than or equal to 8 and was statistically significant (*p* < 0.01). (TIF 26685 kb)

## Abbreviations

AMH: Anti-mullerian hormone
BDNF: Brain-derived neurotrophic factor
CM: Conditioned media
CYP19A1: Cytochrome P450 family 19 subfamily A member 1
E2: Estradiol
EGF: Epidermal growth factor
EGFR: Epidermal growth factor receptor
ELISA: Enzyme-linked immunosorbent assay
FACS: Fluorescence-activated cell sorter
FOXL2: Forkhead box L2
FSH: Follicle-stimulating hormone
FSHR: Follicle-stimulating hormone receptor
hAMSC: Human amniotic mesenchymal stem cell
H&E: Hematoxylin and eosin
hGC: Human granulosa cell
HGF: Hepatocyte growth factor
NOA: Natural ovarian aging
PBS: Phosphate-buffered saline
TGF: Transforming growth factor
